# Recent advances in understanding the effects of T lymphocytes on mucosal barrier function in allergic rhinitis

**DOI:** 10.3389/fimmu.2023.1224129

**Published:** 2023-09-12

**Authors:** Maolin Yang, Liwei Sun, Dongdong Zhu, Cuida Meng, Jichao Sha

**Affiliations:** ^1^ Department of Otolaryngology Head and Neck Surgery, China-Japan Union Hospital of Jilin University, Changchun, China; ^2^ Jilin Provincial Key Laboratory of Precise Diagnosis and Treatment of Upper Airway Allergic Diseases (20190901003JC), Changchun, China

**Keywords:** allergic rhinitis, nasal mucosal barrier, T cells, biological therapy, cytokine

## Abstract

Allergic rhinitis is a non-infectious chronic inflammatory disease of the nasal mucosa that affects T cells and their cytokines. T cells play significant roles in the development of allergic inflammatory diseases by orchestrating mechanisms underlying innate and adaptive immunity. Although many studies on allergic rhinitis have focused on helper T cells, molecular makeup, and pathogenesis-related transduction pathways, pathological mechanisms have not yet been completely explored. Recent studies have suggested that T cell status may play an important role in the interaction between T cells and the nasal mucosal barrier in allergic rhinitis. This study aimed to explore the interactions between T cells and nasal mucosal barriers in allergic rhinitis and to review the therapeutic modalities of pertinent biological agents involving T cells.

## Introduction

Allergic rhinitis (AR) is a non-infectious chronic inflammatory disease of the nasal mucosa. The global prevalence of AR is approximately 10–40% with at least 500 million patients worldwide, accounting for more than 50% of atopic diseases (1, 2). AR is an IgE-mediated type I hypersensitivity response that involves the proliferation and secretion of goblet cells in the nasal mucosa, the tight junctions between epithelial cells, and interactions between various immune cells represented by T lymphocytes, cytokines represented by IL-4, and inflammatory mediators represented by histamine (3).

The Th1/Th2 and Th17/Treg cell balance and other T cell-related parameters have been found to be strongly related to AR (4–6). Advancing research continues to find new aspects of T cell involvement in AR, although the full range of T cell involvement in AR pathogenesis has not been completely elucidated. T cells in AR are currently a hot topic of research because of the recent discovery that their impact on the mucosal barrier influences AR development.

While AR is not life-threatening, it exerts a significant impact on quality of life, emotional state, and economic strain. This paper thus strives to review and summarise current research into the role of the mucosal barrier, the effects of T cells on the nasal mucosa, and the management of biological agents involving T cells.

## Effects of T cells on nasal mucosa

The number of T cell subsets involved in AR, which currently includes Th1, Th2, Th9, Th17, Th22, and regulatory T (Treg) cells, continues to increase owing to ongoing research on this disease. As a non-infectious chronic inflammatory disease of the nasal mucosa, AR is not contagious. Patients with AR experience an immediate reaction characterised by sneezing, rhinorrhoea, and nasal congestion after exposure to intranasally applied allergens, and a late-phase inflammatory response characterised by an influx of eosinophils, basophils, and T cells secreting Th2 cytokines into the nasal mucosa (7). An increasing number of studies have demonstrated a significant influence of T cells on the nasal mucosa of patients with AR. For example, Th2 cells and their cytokines increase epithelial barrier permeability, mucus secretion, and eosinophil infiltration into the nasal mucosa, whereas Th9, Th17, and Th22 cells and their cytokines mainly increase infiltration of eosinophils and secretion of IgE, histamine, and leukotrienes into the nasal mucosa, aggravating inflammatory symptoms. Th1 and Treg cells and their cytokines mainly suppress the inflammatory response mediated by Th2 cells, reducing symptoms in the nasal mucosa.

### 2.1 Structural characteristics and functions of the nasal mucosal barrier

Numerous studies have demonstrated that the mucosal barrier is crucial to successful treatment of infectious and inflammatory illnesses. For example, the intestinal mucosal barrier blocks entry of large antigens from the gut lumen (8). When the loss of intercellular tight junctions and elevated zonulin levels result in increased intestinal mucosal permeability, or when elevated levels of LPS-binding protein (LBP) and soluble CD14 in children with multisystemic inflammatory syndrome (MIS-C) signify loss of gastrointestinal mucosal integrity, the severe acute respiratory syndrome coronavirus 2 (SARS CoV-2) viral antigen can pass through the mucosal barrier and enter the bloodstream, causing antigenemia and other symptoms (9). In AR, the epithelial barrier, nasal epithelial cells that contribute to the nasal mucosal barrier, is the first line of defence in the immune system (10), preventing the entry of pollutants and allergens to a limited extent. Limitations include environmental factors such as air-borne diesel exhaust particles that can damage epithelial tight junctions and increase interepithelial permeability. Increased permeability impairs the epithelial barrier, making it easier for allergens to enter subepithelial tissues (11).

The number of studies on the roles of nasal mucosal membrane in inflammation has increased in recent years. The mucosal layer, consisting of the epithelium with pseudostratified columnar epithelial cells, goblet cells, basal cells, and basement membrane, comprises the three layers of the mucosal barrier in the nasal cavity (12). Nasal mucus, which is mostly produced by the submucosal glands and goblet cells, serves as a crucial protective barrier against the entry of pathogenic microorganisms. Mucus is divided into two layers: an upper gel layer and a lower sol layer close to the cilia, and is composed primarily of mucins, which are mainly encoded by the MUC gene. Mucin synthesis is stimulated by cytokines such as IL-4, IL-9, and IL-13, which lead to increased mucus viscosity, impaired clearance of submucous cilia, mucus build-up in the nasal cavity, and increased nasal congestion (13). The condition of the nasal mucus and its resident microorganisms impact its function and effect in AR. For instance, *Staphylococcus aureus* in the nasal mucus can inhibit the expression of secreted cytokine IL-33 by the nasal epithelium, thereby inhibiting the secretion of Th2 cytokines through the IL-33/ST2 axis and reducing the Th2 cell-dependent inflammatory responses such as AR-associated inflammation of the nasal mucosa (14). T cells, their cytokines, and other inflammatory cells, including mast cells and eosinophils, impact the appearance of the nasal mucosa, including the level of inflammatory cell infiltration and changes in mucosal permeability. The intercellular junctional integrity of linked epithelial cells plays a major role in mucosal barrier strength. Tight junctions (TJs), adherens junctions, and desmosomes are three types of intercellular junctions. TJs contain transmembrane proteins including occludin, tricellulin, claudin family proteins, and junctional adhesion molecules. Claudin family-regulated pore pathways and zonula occludens (ZO)- and occludin-regulated leaky pathways are two transport pathways associated with TJs. The presence of T cells and their cytokines affects the state of tight junctions in the nasal epithelia. Tight junction breakdown opens leaky pathways and lowers transepithelial resistance (TER), increasing the permeability of the epithelial barrier (15). We discovered that the reduced proliferation of basal cells may affect the ability of the epithelium to repair itself, leading to epithelial defects that compromise the integrity of the mucosal barrier and increase the risk of inflammation. Additionally, abnormal basal cell proliferation and differentiation into goblet cells may promote mucus secretion and worsen nasal congestion during inflammation (16). As mentioned above, various mucosal barrier components may play roles in inflammation that vary depending on their current states. Since the mechanisms by which inflammatory factors act on the mucosal barrier are poorly understood, more in-depth research is required to explore additional mucosal barrier components that may play roles in the development of inflammation.

**Figure 1:**
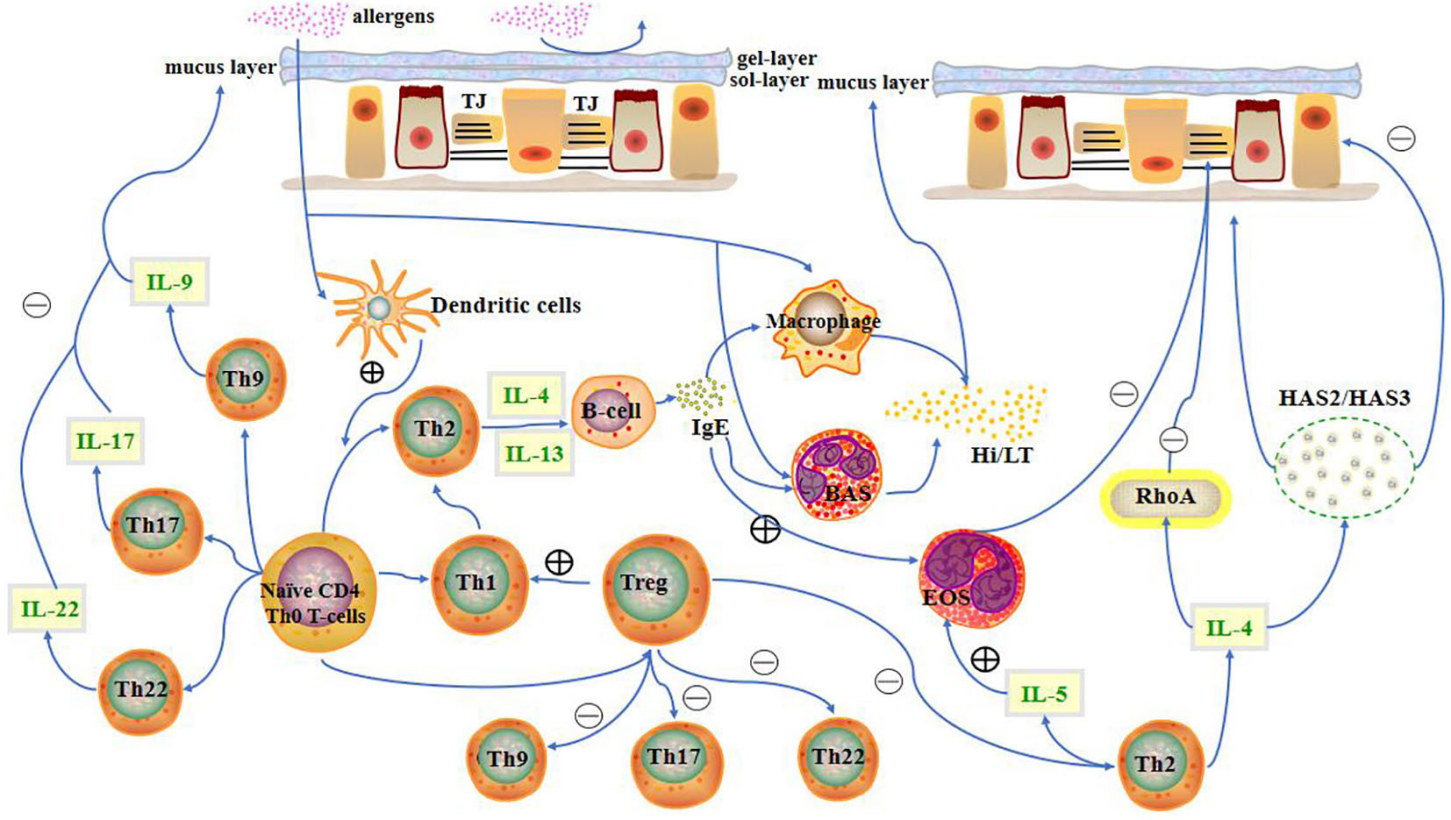
Presence of the nasal mucosal barrier can prevent allergens from entering the body to a limited extent. When allergens cross the mucosal barrier, CD4^+^ T cells differentiate into different T cell subtypes. T cells, along with their cytokines, other immune cells, and inflammatory factors, influence the state of the nasal mucosa. LT, leukotriene; Hi, histamine; HAS, hyaluronic acid synthases; TJ, Tight junction; BAS, basophils; EOS, eosinophils.

A mucosal barrier can block the entry of allergens into the body to a limited extent. When allergens cross the mucosal barrier, they are recognised and presented by dendritic cells to CD4^+^ T cells. Additionally, the interaction between CCL28 and CCR3/CCR10 drives memory CD4^+^ T cell migration into the nasal mucosa (17). CD4^+^ T cells primarily differentiate into Th2 cells upon stimulation with antigens. Th2 cells mainly secrete IL-4, IL-5, and IL-13. IL-4 and IL-13 promote plasma cells to secrete immunoglobulin E (IgE), which in turn promotes histamine and leukotriene secretion by mast cells and basophils. Secretion of these substances promotes smooth muscle contraction in the nose, which increases capillary permeability and glandular secretion, resulting in increased mucus in the nasal mucosa (**Figure 1**).

### 2.2 Effects of Th2 cells on the nasal mucosal barrier

Th2 cells have been linked to pathological alterations in the nasal mucosa of patients with AR. Th2 cells primarily produce IL-4, IL-5, IL-13, and GM-CSF as mediators. RhoA, a small GTPase, is a molecular modulator of TJ homeostasis. IL-4 increases RhoA expression. When Th2 cells from individuals with AR secrete cytokines owing to upregulated RhoA expression, IL-4 may contribute to TJ catabolism. In addition, patients with AR have substantially lower levels of ZO-1 and occludin mRNA expression, which cause TJ catabolism. This improves Fluorescein-dextran isothiocyanate 4kDa (FD4) paracellular passage, lowers TER, and uncovers leakage pathways. Eventually, leakage pathways allow large molecules such as antigens to infiltrate the body and cause inflammation (15). In cases of nasal inflammation, expression of TJ proteins, such as ZO-1 and claudin-1, is frequently downregulated, affecting TJ function, eventually disrupting the epithelial barrier and increasing permeability. There have also been reports of undamaged pseudostratified ciliated columnar epithelium, higher TJ protein expression, lower epithelial permeability, and stronger mucosal barrier defence mechanisms in inflammatory conditions (18). More detailed research is needed to clarify the exact modifications of TJs within the epithelial barrier during inflammation. For Th2 cells, GATA-3 is a crucial transcription factor. The generation of cytokines, such as IL-4, and the differentiation of CD4+ T cells into Th2 cells are regulated by GATA-3 expression (19). One recent study indicates that miR-466a-3p targets GATA-3. In this study, rats with AR transfected with lentiviral miR-466a-3p displayed diminished GATA-3 expression, inhibition of Th2 cell initiation, reduced secretion of Th2 cytokines, and reduced IgE. The number of infiltrating eosinophils in the nasal mucosa of AR mice transfected with lentiviral miR-466a-3p was significantly decreased, and the mice displayed noticeably fewer nasal symptoms, such as scratching and sneezing. This was due to inhibition of Th2 cell initiation and decreased secretion of Th2 cytokines (20). Another study has shown that the Th2 cytokine IL-4 promotes the expression of hyaluronic acid (HA) and the HA synthases (HAS), HAS2 and HAS3, in individuals with AR. While a gradual upregulation of HAS3 promotes the transformation of suprabasal the intermediate progenitor cells (IPCs) into goblet cells, rapid HAS2 upregulation promotes IPC proliferation and basal-to-suprabasal IPC transition. Thus, upregulation of HAS2 and HAS3 in the presence of Th2 cytokines such as IL-4 promotes goblet cell proliferation and mucus secretion, worsening nose congestion in AR (21).

The Th2 cytokine, IL-13, also stimulates the production of inflammatory factors, including granulocyte macrophage colony-stimulating factor (GM-CSF), eotaxin, and the mucin encoded by MUC5AC (a glycoprotein of the mucin superfamily). Additionally, the TLR4/MyD88 pathway controls MUC5AC mRNA expression and generation of inflammatory cytokines (22). AR symptoms worsen with overproduction of mucin and inflammatory cytokines. While LINC00632 inhibits IL-13-mediated induction of inflammatory factors and mucus production, miR-498 exerts a reverse effect. *IL-1RN* produces an IL-1 receptor antagonist (IL-1RA) that interferes with IL-1 signalling by inhibiting nuclear factor κB signalling when IL-1RA binds to the IL-1 receptor. It has been shown that miR-498 negatively regulates *IL-1RN* expression, while LINC00632 positively regulates it. Thus, the LINC00632/miR-498/*IL-1RN* axis controls the synthesis of inflammatory cytokines and mucin in the mucus induced by IL-13, which can reduce AR (23). Additionally, miR-141 controls mucus production via IL-13. The presence of miR-141 influences mucin MUC5AC mRNA translation and basal cell differentiation into goblet cells, indirectly modulating the quantity and growth of goblet cells, and consequently influencing mucus secretion (24). Additionally, production of the Th2 cytokine, IL-5, increases the degree of eosinophil infiltration, whereas IL-5 deficiency decreases both the quantity and intensity of eosinophil infiltration (25). As Th2 cytokines such as IL-4 are secreted less frequently, fewer plasma cells and less IgE are produced. This decreases the release of inflammatory mediators and the invasion of mast cells and eosinophils into the nasal epithelium. Cellular reactions in the nasal mucosa are diminished when the release of inflammatory factors is compromised.

### 2.3 Effects of Th1 cells on the nasal mucosal barrier

The primary cytokines produced by Th1 cells are IL-2, IFN-γ, and TNF-β (25). The amount of IRF1, the transcription factor driving IFN-γ expression, rises when the number of Th1 cells rises, leading to increased IFN-γ secretion and an enhanced Th1 cell response. Upregulation of Bcl2L12, an anti-apoptotic agent, in CD4^+^ T cells damages the apoptotic apparatus. Thus, Bcl2L12 is a critical player in the aetiology of Th2 cell polarisation (26, 27). IRF-1 inhibits Bcl2L12 expression in Th2 cells. As a result, when the Th1 cell response increases, the expression of Bcl2L12 decreases, diminishing the Th2 cell response. From the experimental results, it can be seen that, the serum of FITC-dextran level of AR mice was significantly higher than that of native mice. When wild type AR mice (WT) and CD154-deficient AR mice (KO) were treated with soluble CD83 which is a member of the Ig superfamily, the inhibitory effect of sCD83 on Bcl2L12 disappeared due to the deletion of CD154. So in this experiment, we found that serum FITC-dextran level of KO was significantly higher than that of WT, which showed that serum dextran level can be used as an indicator of nasal mucosal permeability. The inhibitory effect of Bcl2L12 is reduced, the amount of dextran in mouse serum increases, and the permeability of the nasal mucosal epithelial barrier increases in CD154-deficient mice (28). As a result of Th1 cell response-mediated IRF1-dependent inhibition of Bcl2L12 expression, the Th2 cell response is diminished, changing the permeability of the nasal mucosal epithelial membrane and reducing the nasal symptoms of AR (29). In a different experiment, we discovered that adipose tissue-derived stem cells (ASCs) could migrate to the nasal mucosa in a mouse model of AR, inhibit Th2 cell responses by enhancing Th1 cell responses, promote IFN-γ secretion, attenuate AR symptoms, and reduce the inflammatory response of eosinophils in the nasal mucosa (30).

### 2.4 Effects of Treg cells on the nasal mucosal barrier

Increased Th2 cell responses and decreased cytokine levels in Tregs have been observed in rodent models of AR. Pathological alterations in the nasal epithelium in AR may also be influenced by Tregs. Foxp3 is a crucial transcription factor in Tregs. Upregulated Notch2 expression alters the balance of Th1/Th2/Th17 cells in AR mice by boosting Foxp3 expression, which promotes Treg cell differentiation and inhibits the expression of Th2 and Th17 cytokines. The nasal mucosa’s inflammatory reaction eventually ameliorates (31). In our previous study that investigated how human induced pluripotent stem cell (iPSCs)-derived mesenchymal stromal cells (MSCs) affect AR, we discovered that iPSC-MSCs could control the number of other Th cell types by promoting Treg cell proliferation, suppressing the Th2 cell response (32).The increase in the number of Treg cells inhibited the Th2 cell response and reduced the disruption of tight junctions and the promotion of the proliferation and development of goblet cells by Th2 cytokines represented by IL-4, thus protecting the tightness between the barrier epithelial cells of the nasal mucosa, avoiding the overproduction of mucus at the nasal mucosa, and protecting the barrier function of the nasal mucosa, ultimately easing AR symptoms and achieving a therapeutic effect. In another study on AR, 5-hydroxytryptamine (5-HT) was associated with dysfunctional Tregs. In AR, post-degranulation mast cells release 5-HT, which prompts dendritic cells (DC) to release IL-6 and IL-21. These two cytokines encourage the conversion of Treg cells into Th17 cells, which disrupts the balance between Tregs and Th17 cells and worsens AR symptoms.

### 2.5 Effects of Th17 cells on the nasal mucosal barrier

The development of AR also correlates with the number of Th17 cells (33). Additionally, a correlation has been reported between the expression of IL-17 and IL-17RB and the severity of AR symptoms (34–36). In a clinical investigation of IL-17 secreted by Th17 cells, we discovered positive correlations between IL-17 levels and both the number of eosinophils and severity of AR symptoms in patients. The patients’ eosinophil counts and the severity of their symptoms, as measured by the visual analogue scale (VAS), increased with increasing IL-17 levels (35). Therefore, controlling the mismatch between Tregs and Th17 cells may aid in reducing AR symptoms. For instance, while investigating the effectiveness of baicalin as a potential AR treatment, we discovered that baicalin improved nasal mucosal thickness and mucus secretion by correcting the Treg/Th17 cell imbalance and reinforced the mucosal barrier (37).

### 2.6 Effects of Th9, Th22, and γδ T cells on the nasal mucosal barrier

Th9 cells, a novel subtype of Th cells, are responsible for the onset and development of several allergic diseases (38). The development of AR is significantly influenced by Th9 cells and the cytokines they produce, including IL-9. In Chinese patients with AR, mRNA levels of important transcription factors, including interferon regulatory factor 4 (IRF4) and PU.1, increased, and the results of the Rhinoconjunctivitis Quality of Life Questionnaire (RQLQ), VAS scores, and peripheral eosinophil (EOS) counts revealed that IL-9 levels correlated negatively with EOS counts, RQLQ results, and VAS scores. This implies that the severity of AR symptoms increases with increasing strength of the Th9 cell response and the amount of IL-9 released (39). Th9 cell development is aided by IRF4. By lowering the expression of IRF4, fexofenadine and fluticasone propionate decreased the number of Th9 cells *in vivo*. Mast cells are activated by Th9 cells and accumulate during allergic inflammation (40), and histamine secretion by mast cells plays a role in the breakdown of the nasal mucosal barrier. We found that blocking IL-9 inhibited the activity of Th9 cells, relieved nasal mucosal congestion and oedema, and reduced allergic rhinitis and nasal mucosal symptoms (41). Th9 cells exert a significant impact on nasal mucosal lesions in patients with AR; however, the precise mechanism remains unknown and requires further research.

Th22 cells are crucial for AR development. In a clinical investigation, a positive correlation was observed between symptom severity and elevated levels of IL-22 secreted by Th22 cells in patients with AR (42). The presence of IL-22 promotes IgE secretion (43, 44), and IgE in turn stimulates mast cells and basophils to release histamine and leukotrienes. Histamine and leukotrienes stimulate smooth muscle contraction in the nasal cavity, activate glandular secretion, and increase capillary permeability, worsening the symptoms of nasal mucosal inflammation.

T cells can be classified into alphabeta (αβ) and gammadelta (γδ) T cells based on their repertoire of antigen recognition receptors. The nasal mucosa’s indigenous leukocytes are called γδT cells. γδT cells are more prevalent and positively correlated with the quantity of other leukocytes infiltrating the nasal mucosa in patients with AR than in healthy individuals. This implies that (γδ) T cells may be involved in constituting the first line of defence of the nasal mucosal barrier. During pathogen infection or in response to environmental factors, (γδ) T cells are closely related to macrophages and CD8+ cells, and (γδ) T cells can induce or activate macrophage-mediated innate immunity at the nasal mucosal barrier. In addition, (γδ) T cells were positively correlated with the number of eosinophils and mast cells infiltrating the nasal mucosa. (γδ) T cells may alter the secretion of mucus at the nasal mucosa by affecting eosinophils and mast cells, thus affecting the function of the mucosal barrier and the inflammatory symptoms at the nasal mucosa (45). However, the specific mechanism of the effect of (γδ) T cells on the nasal mucosal barrier in allergic rhinitis remains unclear and still needs to be investigated.

Th1, Th2, Treg, Th9, Th17, and Th22 cells play significant roles in the development of nasal mucosal lesions in AR; however, the precise mechanism through which they work, as well as the question of whether other T cells are also involved, require further study (**Table 1**).

**Table 1:** In allergic rhinitis, different types of T cells and the cytokines they secrete affect the state of the nasal mucosal barrier and nasal tissues by altering anatomical structures or cellular components, ultimately aggravating or alleviating nasal symptoms.

T cell	cytokines	effect	Change in the nasal mucosal barrier	references
Th2	IL-4	TJ catabolism	Increased permeability	(15)
		HAS2 and HAS3 upregulation	Stimulation of goblet cell proliferation and mucus secretion	(21)
	IL-13	Secretion of inflammatory factors and mucin	Stimulation of mucus secretion	(22)
	IL-5	the quantity and intensity of eosinophils	Stimulation of mucus secretion	(25)
Th1	IFN-γ	Increased IRF1 expression; Th2 cell response is diminished	Increased permeability of the nasal mucosa’s epithelial barrier	(28)
Treg	IL-10 TGF-β IL-35	Inhibited expression of Th2 and Th17 cytokines.	Eventual alleviation of nasal mucosa’s inflammatory reaction	(31)
Th17	IL-17	Change in number of eosinophils	Nasal mucosal thickness and mucus secretion	(35, 37)
Th9	IL-9	Th9 cell-dependent activation and accumulation of mast cells	Nasal mucosal congestion and oedema	(40, 41)
Th22	IL-22	Secretion of IgE	Contraction of smooth muscles in the nasal cavity, glandular hormone secretion, and increased capillary permeability	(43, 44)

## Biological agent therapy targeting relevant cytokines

Treatment options for allergic rhinitis are increasing because the factors understood to contribute to its pathogenesis are becoming increasingly complex. Traditional drug therapy, biological therapy, specific antigen immunotherapy, combined biological and specific antigen immunotherapy, and non-drug therapies, such as nasal rinses (46) and aerobic exercises (47), are among the available treatment methods. Conventional first-line drugs used in Western medicine for AR include nasal glucocorticoids, antihistamines (48, 49), leukotriene receptor antagonists (50), non-nasal glucocorticoids, mast cell stabilisers (51), decongestants, and nasal anticholinergics (52). Traditional first-line drug therapy is the preferred treatment for AR. For instance, glucocorticoids can be used to treat AR and enhance the condition of the nasal mucosa (53). Antihistamines can reduce inflammatory symptoms in the nasal mucosa by regulating the expression of Th2 cytokines (54). Second-line medications are frequently administered in conjunction with first-line drugs. However, they cannot cure AR; they can only manage the condition’s symptoms. Subcutaneous and sublingual delivery methods are available for immunotherapies that offer potential as cures for rhinitis. Combination therapy with biological agents and specific allergen immunotherapy, such as anti-IL-4 antibodies combined with allergen immunotherapy, has emerged as a novel treatment option for patients who have not responded to immunotherapy (55), and the therapeutic impact of a single immunotherapy is significantly increased by this novel treatment modality. The next section focuses on recently reported biological agent therapies that target relevant cytokines.

### Biological therapeutic agents related to T cells and their cytokines

The TER of both Calu-3 epithelial cells and Primary nasal epithelial cells (pNEC) cultures decreased after three days of growth at the air-liquid interface with conditioned media from Th1 and Th2 cells. Additional *in vitro* research showed that three days of activation with TNF-α and the Th2 cytokines IL-4 and IL-13 decreased TER, increased permeability, and compromised barrier function. Destruction of the mucosa caused by cytokines IL-4, IL-13, and TNF-α secreted by Th1 and Th2 cells was abrogated by pre-treating Calu-3 epithelial cells or pNEC cultures with anti-IL-4R or anti-TNF-α monoclonal antibodies at ALI for 2 h. Our previous study revealed that mice administered with IL-4, IL-13, and TNF-α displayed reduced occludin and ZO-1 expression and increased FD4 permeability than mice administered with intranasal saline. In mice provoked with HDM as opposed to mice triggered with saline, higher IL-4 and TNF concentrations were observed in BAL, indicating allergic airway inflammation. However, pre-treatment with an anti-IL-4 antibody decreased IL-4 levels in the BAL fluid, decreased FD4 permeability, which returned to normal, and stopped albumin leakage. This implies a reduction in TJ destruction by IL-4 and protection and repair of nasal mucosal barrier function by anti-IL-4 antibodies. Disruption of the nasal mucosal barrier and leakage of albumin from pulmonary BAL fluid were both partly prevented by anti-TNF-α antibody therapy (56). In another pre-clinical study that used an anti-TNF-α monoclonal antibody to treat AR, we discovered that infliximab could reduce AR symptoms by lowering local and systemic production of Th2 cytokines, including IL-4, and by reducing IgE levels *in vivo*, thereby reducing the degree of eosinophil infiltration in the nasal mucosa. Thus, an anti-TNF-α monoclonal antibody is potentially therapeutic for the management of AR (57). Both IL-4 and IL-13 receptors are blocked by dupilumab. In clinical trials, the dupilumab group showed a substantially lower incidence of severe treatment deterioration than the placebo group. Additionally, a significant improvement was observed in FEV1 in individuals with concurrent perennial AR from week 2 until week 52. Compared to the placebo group, the dupilumab group showed improvement in the RQLQ(S)+12 sub-scores of nasal complaints at week 52 (58). In another clinical study, patients in the monoclonal antibody group exhibited lower levels of Th2 cytokines (IL-4, IL-5, and IL-13) in their nasal fluid than those in the placebo group. The capacity of CD4^+^ T cells to mediate pro-allergic responses and Th2 cell expansion were inhibited by monoclonal antibodies (REGN1908-1909) against Feld 1 (Felis domesticus allergen 1) in additional *in vitro* studies. As a result, the disruption of the nasal mucosal barrier structure related to Th2 cells and their cytokines, such as the breakdown of TJs and the proliferation and development of goblet cells, is suppressed, and the defence function of the nasal mucosal barrier is strengthened by the tightness of the nasal mucosal barrier and the appropriate amount of mucus. Additionally, because IL-5 and IL-13 are linked to reduced total nasal symptom scores, monoclonal antibodies against these cytokines reduce inflammatory symptoms in the nasal mucosa in individuals with AR (59). Th17 cells and IL-17 are crucial for AR development and progression, and IL-23 is essential for Th17 cell responses and IL-17 secretion. Th17 cell-associated biological agents significantly affect AR treatment. One study used anti-IL-23 and anti-IL-17 antibodies to treat mice with AR. Anti-IL-23 antibody treatment prevented neutrophil, eosinophil, and mast cell infiltration into the nasal mucosa in mice. Infiltration of neutrophils and eosinophils into the nasal mucosa was blocked using anti-IL-17 antibody treatment. Both treatments reduced symptoms in the nasal mucosa, such as rubbing and sneezing; however, the anti-IL-23 antibody was more successful (60). Eosinophils and mast cells in the nasal mucosa can disrupt the integrity of the nasal mucosal epithelium and alter the permeability of the epithelium. Anti-IL-17 and anti-IL-23 antibodies protect the defence function of the nasal mucosal barrier by inhibiting the extent of their infiltration at the nasal mucosa (56).

## Conclusion

The effect of T cells interaction with the nasal mucosal barrier on AR development and progression varied depending on the type of T cell cytokine that dominates in the affected tissue. Biological agents targeting T cell cytokines are thus gradually becoming important options for AR treatment. Future studies will continue to explore the specific mechanisms by which interactions between T cells and the nasal mucosal barrier or other T cell types, as well as the types of biological agents targeting T cell cytokines, impact AR, which will provide great opportunities to refine the path.

## Author contributions

JS and MY conceived the idea of the study. LS and MY searched literature. CM and DZ collected and analysed data. MY and JS wrote the paper. All authors discussed the results and revised the manuscript. All authors contributed to the article and approved the submitted version.
